# Nursing case management for people with hypertension: A randomized controlled trial protocol

**DOI:** 10.1097/MD.0000000000023850

**Published:** 2020-12-24

**Authors:** Chunjing Song, Xianhong Li, Xueling Ning, Shiqiang Song

**Affiliations:** Department of Urology, People's Hospital of Chengyang District, Qingdao, China.

**Keywords:** nursing case management, hypertension, blood pressure, protocol

## Abstract

**Objective:**

To explore the effect of management of nursing case on blood pressure control in hypertension patients.

**Method:**

This is a randomized controlled study which will be carried out from May 2021 to May 2022. The experiment was granted through the Research Ethics Committee of the People's Hospital of Chengyang District (03982808). Our research includes 200 patients. Patients who meet the following conditions will be included in this experiment: the patients aged 18 to 60 years; the patients had the diagnosis of hypertension; and the urban residents. While patients with the following conditions will be excluded: having renal failure, liver failure, heart and respiratory failure; and known pregnancy. Primary result is blood pressure, while secondary results are treatment compliance, waist circumference, body mass index (BMI), type and number of antihypertensive agents used, and the existence of metabolic and cardiovascular comorbidities.

**Results:**

Table 1 shows the clinical outcomes between the two groups.

**Conclusion:**

Nursing case management is effective to improve the prognosis of hypertension patients.

## 1 Introduction

Hypertension is one of the cause of death worldwide, which is preventable.^[1,2]^ It is also a significant risk factor for myocardial infarction, heart failure, stroke, as well as other serious renal and cardiovascular diseases.^[3–5]^ The incidence rate of hypertension rises with the age of adults. It is reported that 36% of the adults aged 40 to 64 suffer from hypertension; among adults aged 65 and above, the proportion has increased to 70%.^[6,7]^ It has become a serious problem of public health. Since the hypertension is asymptomatic, its detection and control remains a challenge. The hypertension patients are managed via the primary health care provider.^[8]^ Nevertheless, although the progress has been made in the management of chronic diseases, the hypertensive patients who receive regular treatment from primary care providers do not meet their targets of blood pressure.

In recent years, more and more researches begin to pay attention to the significant role of the management of nursing case in treating hypertension.^[9,10]^ It requires a complex care, involving major lifestyle changes such as adherence to medication, reduced salt intake, the measurement of blood pressure and exercise. Nevertheless, the hypertension patients have poor self-management behaviors. The self-care and self-efficacy behavior of uncontrolled hypertension patients are lower. Case management is a kind of healthcare strategy that determines patients at high risk, prevents complications and disease progression, and promotes the patients participation in self-care. Other targets involve caring for the perspectives and needs of patients, developing personalized care programs, improving the quality of health care, and decreasing decentralized patient care. The former researches have suggested that management of case may have a positive effect on hypertension.^[11–13]^ In addition, it can increase the knowledge about the disease; adhere to the treatment plans and help the patients improve their own lifestyle. Although it has achieved positive results in the case management of chronic disease, it has not been applied in patients with hypertension. Hence, we conduct the randomized controlled study protocol to explore the effect of management of nursing case on blood pressure control in hypertension patients.

## 2 Materials and methods

This is a randomized controlled study which will be carried out from May 2021 to May 2022 at the People's Hospital of Chengyang District. The experiment was granted through the Research Ethics Committee of the People's Hospital of Chengyang District (03982808) and recorded in research registry (researchregistry6244).

### 2.1 Inclusion criteria and exclusion criteria

Patients who meet the following conditions will be included in this experiment: the patients aged 18 to 60 years; the patients had the diagnosis of hypertension; and the urban residents. While patients with the following conditions will be excluded: having renal failure, liver failure, heart and respiratory failure; and known pregnancy. All the patients are randomly assigned to the random number through utilizing a random-number table, and the result of distribution is kept in a random envelope and is invisible. All the patients are randomly divided to the control group and study group, and there are 100 patients in each group.

### 2.2 Nursing case management

The nursing standards of the control group are as follows: renewal of prescriptions in meetings, free distribution of hypertension medication, and the monitor of blood pressure every 2 months, nursing and medical appointments, and consultation with psychologists and nutritionists based on the needs of patients.

In intervention group, patients are given management of nursing case. From the existing management activities, the arrangements are as follows: telephone contacts, nursing consultations, personal health education activities, and home visits. The nursing consultations are implemented every 6 months. The purpose of the consultation is to gather information that can be utilized to draft personal care plans and to set mutually agreed targets. The consultation lasts about an hour, involving the targeted health education, the measurement of waist circumference and blood pressure, and the calculation of BMI. Telephone contact is conducted every 1 month to reassess the healthcare plans of patients and remind the patients to consult the agendas in a timely manner. WeChat is a kind of instant messaging tool, which allows the voice calls through using the mobile phone, and it is also utilized for communication. Each telephone meeting lasts about 10 min. In the process of home visits, the case manager will observe the home environment, for instance, the living conditions and family's interaction. They offer the health education, check the weight of patient and their blood pressure, and then review the targets and medical plans. All the verbal instructions will be recorded and the patients will be provided the copy for consultation if needed. For the home visits, it lasts about 45 min. And the group activities contain the interactive activities and informational lectures. The focus of these activities is to develop healthy habits. The theme of educational activities is selected according to patients’ main needs. The activities of collective health education are carried out in community space. These group activities last about 1 h. Personalized educational activities are offered in the process of nursing consultation, telephone consultation, and home visit. All information acquired in the process of nursing management will be recorded.

### 2.3 Outcomes

Primary result is blood pressure, while secondary results are treatment compliance, waist circumference, BMI, type and number of antihypertensive agents used, and the existence of metabolic and cardiovascular comorbidities.

### 2.4 Statistical analysis

The analysis of all the data are conducted with the software of IBM SPSS Statistics for Windows, version 20 (IBM Corp, Armonk, NY). Afterwards, all the data acquired are represented through the appropriate characteristics, for example, standard deviation, and mean, median as well as percentage. And independent *t* tests and χ^2^-tests are respectively utilized to analyze the categorical variable and continuous variable. *P* value < .05 indicates that there is statistical significance.

## 3 Result

Table 1 shows the clinical outcomes between the two groups.

**Table 1:** The clinical outcomes between the two groups.

Outcomes	Study group	(n = 100)	Control group (n = 100)	*P*
Systolic blood pressure				
Diastolic blood pressure				
Mean arterial pressure				
Waist circumference				
Body mass index				
Quality of life score				

## 4 Discussion

Hypertension is the most significant risk factor for disability and death worldwide, which affects more than one billion people and causes ∼9.4 million deaths each year.^[14]^ On the basis of a report by the World Health Organization, hypertension is the single most significant risk factor, which accounts for 13% of global mortality. Human hypertension may be the result of lifestyle and genetic factors.^[15,16]^ The current evidence-based treatment for the hypertension is a key intervention measure to reduce the incidence rate and mortality of cardiovascular diseases. Researches have determined a variety of barriers to the control of hypertension in routine care that are composed of factors related to patients, physicians, healthcare system, and healthcare services.

People with lower income and education levels are more likely to be insufficiently physically active, which predisposes them to the risk of complications associated with chronic diseases, particularly the hypertension.^[17]^ In contrast, people with higher educational and economic levels tend to be more effective at controlling the levels of blood pressure. Therefore, it is essential to consider the effect of these variables and then incorporate these variables into the development of nursing planning and educational activities for hypertension patients. Case management can be utilized for this objective by providing a personalized plan based on each person's needs.

## 5 Conclusion

Nursing case management is effective to improve the prognosis of hypertension patients.

## Author contributions

Shiqiang Song designs the protocol. Xianhong Li reviews the protocol. Xueling Ning performs the data collection. Chunjing Song finishes the manuscript. All of the authors approved the submission.

**Conceptualization:** Xianhong Li.

**Data curation:** Xianhong Li.

**Funding acquisition:** Shiqiang Song.

**Investigation:** Xueling Ning.

**Methodology:** Xueling Ning.

**Writing – original draft:** Chunjing Song.
